# Editorial: The cross-talk between cancer cells and immune cells in the tumor microenvironment

**DOI:** 10.3389/fmed.2023.1328045

**Published:** 2023-11-16

**Authors:** Tao Li, Bo Li, Qianqian Song, Wenjie Zheng

**Affiliations:** ^1^Department of Neurosurgery, Tianjin Medical University General Hospital, Tianjin, China; ^2^Department of Orthopedics, Beijing Luhe Hospital, Capital Medical University, Beijing, China; ^3^Department of Cancer Biology, Wake Forest University School of Medicine, Winston-Salem, NC, United States; ^4^Research Center of Clinical Medicine, Affiliated Hospital of Nantong University, Nantong, China

**Keywords:** tumor microenvironment, cancer cell, immune cell, prognostic marker, immunotherapy

The tumor microenvironment (TME) refers to a dynamic environment around the tumor, where tumor cells interact with the host stroma including diverse immune cell types, cancer-associated fibroblasts, endothelial cells, pericytes, and various additional tissue-resident cell types (1, 2). TME is a critical regulator of cancer progression involving in tumor growth, invasion, apoptosis, angiogenesis, drug resistance, and immune evasion (3–5). For instance, cancer cells hijack host stroma to support their growth and evade immune surveillance. Numerous studies have identified cytokines, extracellular vesicles, and metabolites secreted by cancer cells that contribute to the remodeling of an immunosuppressive TME. Decoding the mechanisms that govern the interplay between cancer cells and immune cells is essential to identify predictive and prognostic biomarkers and improve the efficacy of cancer immunotherapies. The articles on this topic collectively illustrate the intricate interplay between the TME, autophagy, biomarkers, and immune cell populations, and their potential roles in cancer progression, prognosis, and immunotherapeutic strategies.

The role of autophagy in cancer is highly intricate and frequently paradoxical. Present research is increasingly focused on the significance of autophagy within the tumor microenvironment. In their review, Yang et al. have shed light on the intricate interplay between autophagy and immune cells in the tumor immune microenvironment. Autophagy can influence tumor immunity through diverse mechanisms involving antigen presentation and the development of immune cells. Autophagy can affect tumor immunity in various ways via antigen presentation and immune cell development. In certain types of immune cells, reducing autophagy may promote an anti-cancer response. However, in other tumor infiltrating immune cells, increased autophagy levels can serve as an effective anti-cancer mechanism. The authors propose that target specific autophagy enhancers could function along with immunogenic chemotherapeutics or immune checkpoint blockade to elevate the efficacy of cancer immunotherapy.

SMAD4 plays a critical role in antitumor immunoregulation, the importance of this tumor suppressor gene in the prognosis and immune response in Hypopharyngeal Carcinoma (HPC) remains unclear. Song et al. conducted a study to investigate the significance of SMAD4 in Hypopharyngeal Carcinoma (HPC) by assessing its expression in HPC tissues and its potential prognostic value. Additionally, they examined the correlation between intratumoral CD8^+^ T cells and peritumoral CD15^+^ neutrophils with SMAD4 levels to determine the impact of SMAD4 on the immune response in HPC. The findings, based on a cohort of 97 HPC patients, suggest that SMAD4 can serve as a valuable predictive biomarker in HPC and may also hold promise as a target for immunotherapy.

Li et al. developed and validated a prognostic model for gastric cancer (GC) using Neutrophil Extracellular Traps Genes. This model accurately predicts the prognosis of GC patients and offers a comprehensive analysis of the role of NETs in the tumor microenvironment, clinicopathological characteristics, and patient outcomes. The NETs-score risk model provides a basis for better prognosis and therapy outcomes in GC patients.

Recent studies have suggested that the presence of tumor-infiltrating lymphocytes (TIL) and tumor-associated macrophages (TAM) could serve as potential indicators for predicting the prognosis of patients with head and neck cancer. De Virgilio et al. presented an analysis of TIL and TAM as potential prognostic factors for neck lymph node metastasis in patients with major salivary gland carcinomas. The study, consisting of 25 cases, is considered an initial exploration in the quest for potential prognostic markers. The findings from this research suggest that an elevated density of certain subpopulations of TIL and TAM is associated with an increased probability of neck metastasis.

## Author contributions

TL: Writing—original draft. BL: Writing—review & editing. QS: Writing—review & editing. WZ: Writing—review & editing.

## References

[1] De Visser KE Joyce JA. The evolving tumor microenvironment: From cancer initiation to metastatic outgrowth. Cancer Cell. (2023) 41:374–403. 10.1016/j.ccell.2023.02.016 36917948

[2] Jiang Y Wang C Zhou S. Targeting tumor microenvironment in ovarian cancer: premise and promise. BBA Rev. Cancer. (2020) 1873:188361. 10.1016/j.bbcan.2020.188361 32234508

[3] Khalaf K Hana D Chou JT Singh C Mackiewicz A Kaczmarek M. Aspects of the tumor microenvironment involved in immune resistance and drug resistance. Front Immunol. (2021) 12:656364. 10.3389/fimmu.2021.656364 34122412 PMC8190405

[4] Jiang X Wang J Deng X Xiong F Zhang S Gong Z. The role of microenvironment in tumor angiogenesis. J. Exp. Clin. Cancer Res. (2020) 39:1–19. 10.1186/s13046-020-01709-5 32993787 PMC7526376

[5] Yaacoub K Pedeux R Tarte K Guillaudeux T. Role of the tumor microenvironment in regulating apoptosis and cancer progression. Cancer Lett. (2016) 378:150–9. 10.1016/j.canlet.2016.05.012 27224890

